# Bergeyella zoohelcum Bacteremia from Therapy Dog Kisses

**DOI:** 10.7759/cureus.4494

**Published:** 2019-04-18

**Authors:** Shorabh Sharma, Hernando Salazar, Sapna Sharma, M. Farhan Nasser, Michelle Dahdouh

**Affiliations:** 1 Internal Medicine, St. Barnabas Hospital Health System, Bronx, USA; 2 Internal Medicine, Mahatma Gandhi Mission Institute of Health Sciences, Navi Mumbai, USA; 3 Vascular Medicine, Cleveland Clinic, Cleveland, USA; 4 Infectious Disease, St. Barnabas Hospital Health System, Bronx, USA

**Keywords:** bacteremia, bergeyella, bergeyella zoohelcum, aids, zoonoses

## Abstract

Bergeyella (B.) zoohelcum is a non-motile, aerobic, gram-negative rod, with only a few cases in the literature. Most of the human infections are related to dog or cat bites; however, there are also reports related to the ingestion of food prepared with goat’s blood. We present a case of Bergeyella zoohelcum bacteremia in a patient with acquired immunodeficiency syndrome (AIDS) following close contact with their service dog. To the best of our knowledge, this is the first case of B. zoohelcum bacteremia in an AIDS patient.

## Introduction

Bergeyella (B.) zoohelcum (formerly known as Weeksella) is a non-motile, aerobic, gram-negative rod. It is a rarely reported zoonotic pathogen with only a few cases in the literature. It has been frequently isolated from the upper respiratory tract of cats, dogs, and other mammals [1]. Most cases are related to dog or cat bites [2-3], but there have also been cases related to the ingestion of food prepared with goat’s blood [4] and in patients with debilitating underlying diseases. We present a case of Bergeyella zoohelcum bacteremia in a patient with acquired immunodeficiency syndrome (AIDS) after contact with her service dog.

## Case presentation

A 43-year-old, male-to-female, transgender patient with an extensive medical history significant for AIDS on antiretroviral therapy, end-stage renal disease (ESRD) on hemodialysis, pulmonary embolism, heart failure with reduced ejection fraction with automated implantable cardioverter defibrillator (AICD) in place, and a history of recurrent ventricular and supraventricular tachycardia with prior ablation presented to our hospital reporting dizziness and several episodes of non-bloody, non-bilious vomiting with decreased appetite since the past few days. She lived with her dog and denied abusing alcohol or any other illicit drugs, with no recent travel history. She did not report any exposure to farm animals and did not have any recent travel history. She was found to have supraventricular tachycardia, which terminated with adenosine. She was afebrile, with a heart rate of 123 and blood pressure (BP) of 124/89, and was saturating 96% on room air. The initial exam was notable for tachypnea without accessory muscle use and tachycardia with a regular rhythm. Labs were remarkable for a high anion gap metabolic acidosis with a lactate of 9 and blood urea nitrogen (BUN) and creatinine of 56 and 9.2, respectively. She was admitted to the telemetry service for presyncope, likely due to the supraventricular tachycardia (SVT). Her lactic acidosis was presumed to stem from hypoperfusion in the setting of her heart failure and arrhythmia. Her AICD was interrogated, which revealed normal device function with no events since her ablation.

The blood culture obtained grew gram-negative bacilli and infectious diseases (ID) were consulted. She was started on levofloxacin and meropenem to cover for Bordetella, Legionella, Brucella, Actinobacillus, Campylobacter, Francisella, Helicobacter, and Pasteurella, among other organisms. The gram-negative bacilli were not growing on MacConkey or Chocolate plates and could not be identified by the microbiology lab. The organism was sensitive to Levaquin, and the patient was eventually discharged on the latter. The specimen was sent to the department of health, where the organism was identified as Bergeyella zoohelcum.

## Discussion

Bergeyella zoohelcum (Greek for animal + wound) is a gram-negative rod that is part of the normal oral microbiota from cats, dogs, and other animals such as piglets [5].

It was formerly known as Weeksella zoohelcum Centers for Disease Control and Prevention (CDC) group IIj but later moved to the new genus Bergeyella. This microorganism grows well in blood agar but most colonies do not grow well in MacConkeys agar. The colonies are circular and very sticky, which makes them difficult to remove from solid media [6]. Some strains of B. zoohelcum only grow on chocolate agar and are biochemically inactive, thus preventing them from recovery. Identification is challenging and there have been cases of misidentification [7].

It is a non-fermentative bacillus but differs from others in being susceptible to penicillin. It is oxidase positive, catalase positive, and indole positive, and differs from Weeksella virosa by its ability to produce urease and being resistant to polymyxin [8].

Most of the reported cases have been related to bites from dogs [2-3], cats [9], Siberian tiger [10], or contact with these animals [6,11-12]. Some colonies have been isolated frequently during routine analyses of food; therefore, oral transmission can be possible. Beltran et al. reported the case of a 44-year-old woman with no significant medical history with B. zoohelcum bacteremia after ingesting food prepared with goat’s blood [4]. Chen et al. reported a case of B. zoohelcum bacteremia in a patient with endocarditis with no clear exposures and no underlying conditions.

Commonly reported infections with B. zoohelcum include cellulitis, abscesses, diarrhea, lymphangitis, and endocarditis [13-14], bacteremia, pneumonia [15], tenosynovitis [10], and bed sore infections [12]. In terms of underlying conditions in the presented cases, Lin et al. reported a case of B. zoohelcum bacteremia in a 73-year-old man with a medical history of liver cirrhosis, hepatitis C, bladder carcinoma, stasis dermatitis, skin graft, and recurrent cellulitis [6]. Noel et al. reported a case of B. zoohelcum bacteremia in an 80-year-old woman with a history of diabetes mellitus and bedsores [12]. Kivinen et al. reported a case of B. zoohelcum cellulitis and bacteremia in a 77-year-old woman with a history of Alzheimer, diabetes mellitus, hypertension, pernicious anemia, heart failure, and polymyalgia rheumatica on chronic steroids [11]. Sohn et al. reported a case of B. zoohelcum endocarditis in a 47-year-old man with a history of paroxysmal supraventricular tachycardia [14].

Our patient had a history of AIDS on antiretroviral therapy, ESRD on hemodialysis, severe systolic heart failure with AICD, and supraventricular tachycardia. This is the first case reported of B. zoohelcum infection in a patient with AIDS. In this case, there was no bite but contact with a dog. Compared with the above cases, our patient shared heart failure, supraventricular tachycardia, and immunosuppression as comorbidities. It is not clear if these conditions are associated with an increased risk for B. zoohelcum infection and if so, what are the mechanisms involved in its development.

B. zoohelcum has shown to be susceptible to β-lactam antibiotics, including penicillin, chloramphenicol, and fluoroquinolones, and are variable in susceptibility to tetracycline and trimethoprim-sulfamethoxazole. In all of the reported cases, treatment was curative, the medications used include crystal penicillin, oxacillin, amoxicillin-clavulanate, ampicillin-sulbactam, cefalexin, oxacillin, ceftriaxone, cefotaxime, ciprofloxacin, and, in our case, levofloxacin and meropenem (Table 1).

**Table 1:** Literature review of Bergeyella zoohelcum infections in humans DM: Diabetes mellitus; HTN: Hypertension; IV: Intravenous; AIDS: Acquired immunodeficiency syndrome; ESRD: End-stage renal disease; AICD: Automated implantable cardioverter defibrillator

Reference	Age (years)/ gender	Exposure	Underlying conditions	Presentation	Treatment	Outcome
Chen et al [13]	27 F	None	None	Bacteremia, endocarditis	Cefuroxime 1.5 gm every 8 hr for 6 weeks Mitral and aortic valve replacement, tricuspid valvuloplasty	Recovered
Montejo et al [2]	33 M	Dog bite	None	Cellulitis, bacteremia	Amoxicillin-clavulanate 1 gm every 8 hr for 10 days	Recovered
Yi et al [3]	22-month-old M	Dog bite	None	Cellulitis, abscess	First day: amoxicillin Second day: ceftriaxone 50 mg/kg a day plus clindamycin 10 mg/kg every 8 hr Third day: amoxicillin-clavulanate 45 mg/kg divided into 2 doses a day to complete 10 days	Recovered
Beltran et al [4]	44 F	Dish made of goat’s blood	None	Diarrhea, bacteremia	Ciprofloxacin, initially IV then oral	Recovered
Shukla et al [9]	60 F	Cat bite	None	Cellulitis, lymphangitis	First day: amoxicillin-clavulanate Second day: ampicillin-sulbactam 3 gm every 6 hours to complete 1 week	Recovered
Kivinen et al [11]	77 F	Cat contact	Alzheimer, DM, HTN, pernicious anemia, heart failure, polymyalgia rheumatica on chronic steroids	Cellulitis, bacteremia	Cefuroxime 1.5 gm every 8 hrs for 6 days Cefalexin 750 mg 3 times a day for 10 days Cefalexin 750 mg 2 times a day for 15 more days	Recovered
Lin et al [6]	73 M	Dog contact	Liver cirrhosis, hepatitis C, bladder cancer, recurrent cellulitis, stasis dermatitis	Cellulitis, bacteremia	Crystal penicillin G 3 million units every 6 hours Then Oxacillin 2 gm every 6 hours Then Cefazoline 1 gm every 8 hours + gentamycin 80 mg every 12 hours Total of 14 days	Recovered
Isotalo et al [10]	35 M	Siberian tiger bite	None	Tenosynovitis	Cefazoline IV 1 gm every 8 hrs + gentamycin 80 mg every 8 hours for 3 days Amoxicillin-clavulanate 500 mg every 8 hours for 7 days	Recovered
Noell et al [12]	80 F	Cat contact	DM	Bedsore infection	Cefotaxime	Recovered
Sohn et al [14]	47 M	None	Paroxysmal supraventricular tachycardia	Endocarditis	Day 1–3: ceftriaxone, ampicillin, and gentamicin Day 4–32: piperacillin/ tazobactam and amikacin Day 33-36: ampicillin/sulbactam	Recovered
This case	43 transgender M to F	Dog contact	AIDS on antiretroviral therapy, ESRD on hemodialysis, severe systolic heart failure with AICD and supraventricular tachycardia	Bacteremia	Meropenem-levofloxacin IV for 3 days. Discharged on levofloxacin oral to complete 10 days	Recovered

## Conclusions

Bergeyella zoohelcum is a rare cause of bacteremia usually seen in patients exposed to cats and dogs. The role of immunosuppression and chronic illness is not well-established. Physicians should suspect this pathogen in patients with gram-negative bacilli bacteremia with a history of exposure to these animals, especially if the regular cultures do not provide identification. Amoxicillin-clavulanate and ampicillin-sulbactam seem to be an effective initial approach in patients with a history of animal bites.
